# MAP2 immunoreactivity deficit is conserved across the cerebral cortex within individuals with schizophrenia

**DOI:** 10.1038/s41537-019-0081-0

**Published:** 2019-08-28

**Authors:** Rebecca DeGiosio, Ryan M. Kelly, Adam M. DeDionisio, Jason T. Newman, Kenneth N. Fish, Allan R. Sampson, David A. Lewis, Robert A. Sweet

**Affiliations:** 1 University of Pittsburgh Department of Psychiatry, Pittsburgh, PA USA; 2 University of Pittsburgh Department of Statistics, Pittsburgh, PA USA; 3 University of Pittsburgh Department of Neurology, Pittsburgh, PA USA

**Keywords:** Cellular neuroscience, Schizophrenia

## Abstract

Several postmortem studies have reported lower levels of immunoreactivity (IR) for microtubule-associated protein 2 (MAP2) in several cortical regions of individuals with schizophrenia (SZ). However, whether this effect is conserved across multiple brain areas within an individual with SZ or if it is regionally-specific remains unclear. We characterized patterns of MAP2-IR across three cortical regions at different levels of the rostral-caudal axis within individual subjects with and without SZ. MAP2-IR levels were measured in deep layer 3 of dorsolateral prefrontal cortex (DLPFC), lateral intraparietal cortex (LIP), and primary visual cortex (V1). Postmortem tissue containing each cortical region was derived from 20 pairs of SZ subjects and nonpsychiatric comparison (NPC) subjects matched perfectly for sex, and as closely as possible for age and postmortem interval. MAP2-IR was assessed by quantitative fluorescence microscopy. We observed significantly lower levels of MAP2-IR in SZ subjects relative to NPC subjects, without a significant region by diagnosis interaction. Logs of the within-pair ratios (SZ:NPC) of MAP2-IR were significantly correlated across the three regions. These findings demonstrate that MAP2-IR deficits in SZ are consistent across three neocortical regions within individual subjects. This pattern of MAP2-IR deficit has implications for therapeutic development and future investigations of MAP2 pathology in SZ.

## Introduction

Microtubule-associated proteins (MAPs), a group of proteins critical to cytoskeletal function, regulate cellular development and maintenance by way of microtubule stabilization/bundling, recruitment of signaling molecules, and modulation of microtubule-mediated cellular transport.^1^ MAP2, a MAP family member which is expressed mainly in neuronal dendrites,^2^ frequently exhibits lower levels of immunoreactivity (IR) in the postmortem tissue of subjects with schizophrenia (SZ) when compared with nonpsychiatric comparison (NPC) subjects. This has been observed in the subiculum,^3,4^ the olfactory bulbs,^5^ entorhinal cortex,^3^ and Brodmann areas 9, 32, and 41.^6–8^ These deficits in MAP2-IR do not seem to result from changes in neuron number, MAP2 mRNA expression, or protein levels,^7,9,10^ but instead seem suggestive of a posttranslational modification (PTM) to the protein. This robust finding fits into a larger theme of cytoskeletal dysregulation in SZ; abnormalities in cytoskeletal organization networks have become increasingly implicated in SZ pathogenesis through the evidence of large-scale genomic studies, proteomic analyses, and immunohistochemical assays.^11–13^ However, because prior studies of MAP2-IR in SZ have examined either a single brain area, or two physically proximate brain areas, it remains unclear whether lower MAP2-IR is regionally specific or is present across the cerebral cortex within individuals with SZ. Similarly, prior studies have not tested whether the magnitude of MAP2-IR deficits are consistent across regions within subjects, although finding that MAP2-IR deficits are conserved across cortical regions within individuals with SZ would motivate future studies of the mechanisms surrounding MAP2 pathology with the goal of developing therapeutic intervention strategies.

**Fig. 1:**
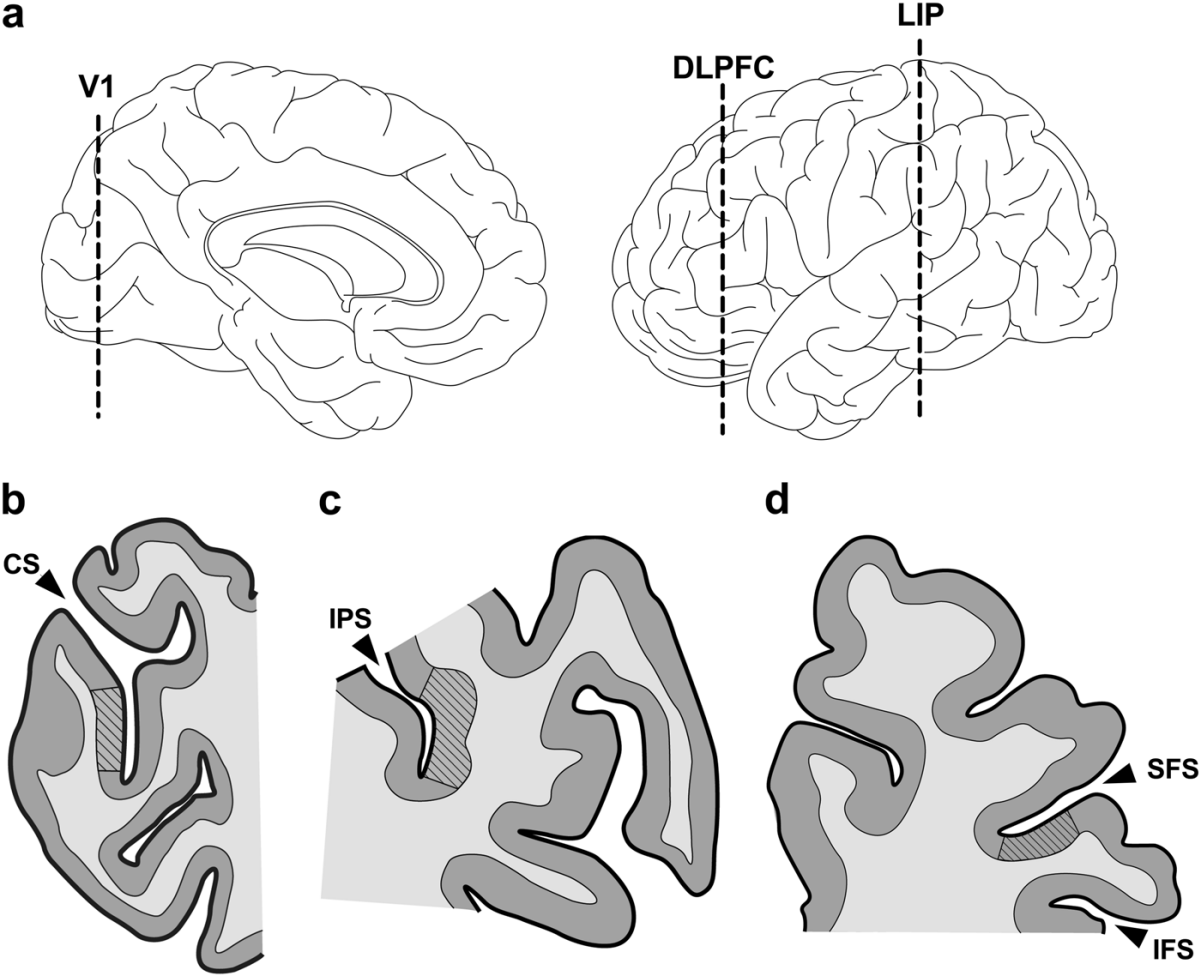
Components of the visuospatial working memory pathway represent the rostro–caudal cortical axis. **a** Rostro–caudal position of human V1, DLPFC, and LIP in medial (left) and lateral (right) view. **b**–**d** Representative tissue sections containing V1 (**b**), LIP (**c**), and DLPFC (**d**). For the present study, coronal blocks containing the calcarine sulcus (CS), intraparietal sulcus (IPS), or middle frontal gyrus (bordered by the superior/inferior frontal sulci [SFS/IFS]) were sliced to derive tissue sections containing V1 (BA17), LIP (BA7), and DLPFC (BA46), respectively. Regions of interest (striped areas) were defined by cytoarchitectonic criteria (see text)

Herein, we characterize within-subject MAP2-IR in SZ across three regions located in separate lobes of the cerebral cortex. MAP2-IR was measured in dorsolateral prefrontal cortex (DLPFC; BA46), lateral intraparietal cortex (LIP; BA7), and primary visual cortex (V1; BA17) using quantitative confocal fluorescent microscopy. These cortical regions are dispersed across the rostral–caudal axis (Fig. 1a), are collectively implicated in visuospatial working memory^14,15^ and show functional impairment in SZ.^16–18^ We observed significantly lower levels of MAP2-IR in SZ subjects compared with NPC subjects. There was no interaction between region and diagnosis, indicating that the degree of IR deficit did not differ significantly between regions. Within-pair ratios (SZ:NPC) of MAP2-IR were significantly correlated across regions. These data indicate that within individuals with SZ, MAP2-IR deficits can exist across multiple cortical areas.

## Results

**Fig. 2:**
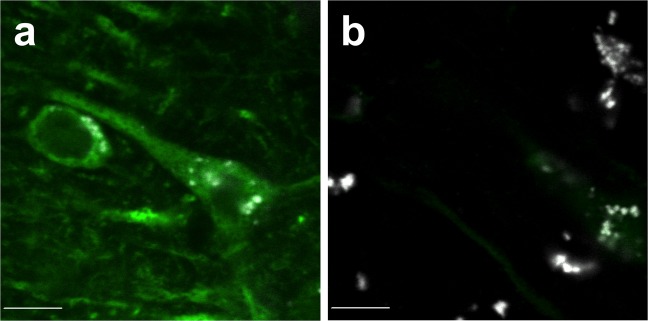
Micrographs of MAP2-IR in a representative pair of subjects. Micrographs are from a subject pair in LIP (filled circle in Fig. 3d). MAP2-IR (green) is significantly reduced in SZ subject (**b**) compared with matched NPC subject (**a**) after accounting for lipofuscin auto-fluorescence (grayscale). This difference is representative of the average 69.3% reduction in linear MAP2-IR value within LIP between diagnostic groups (shown as a reduction of 22.8% in log_e_[MAP2-IR] in Fig. 3a). Scale bars = 10 µm

**Fig. 3:**
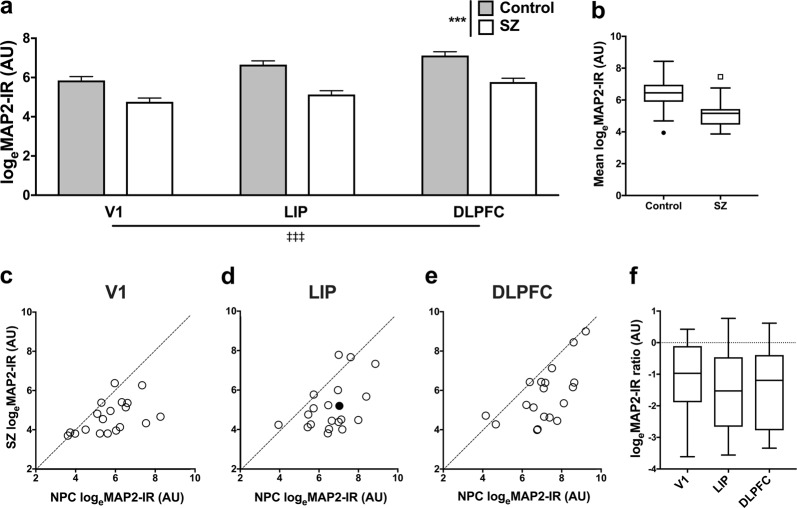
MAP2-IR is reduced uniformly in V1, LIP, and DLPFC of SZ patients compared with matched NPC subjects. **a** Estimated marginal means of log_e_(MAP2-IR) grayscale intensity levels by diagnostic group and cortical region. MAP2-IR differed significantly between groups and between regions, but there was not a significant diagnosis by region effect. *N* = V1: 19/20 (SZ/NPC); LIP: 20/20; DLPFC: 20/19. Error bars = SEM. ***p < 0.001 (diagnosis effect).  *p* < 0.001 (region effect). **b** Tukey boxplots by diagnostic group of log_e_(MAP2-IR) levels averaged across region. *N* = 18 subjects per group. **c**–**e** Control and SZ log_e_(MAP2-IR) levels in V1 (**c**), LIP (**d**), and DLPFC (**e**) shown by pair relative to a y = x line of no change. *N* = 18 pairs. Exemplary micrographs from a pair in LIP (filled dot in **d**) are shown in Fig. 2. **f** Tukey boxplots of log_e_(MAP2-IR) pairwise ratios (SZ:NPC) by region. *N* = 18 pairs per region. AU = arbitrary units

We initially sought to determine whether MAP2-IR (as defined by greyscale intensity [see “Methods” section]) was lower in SZ subjects as predicted. In addition, we asked whether MAP2-IR differed across cortical regions. Qualitatively, images from SZ tissue frequently exhibited a paucity of MAP2 labeling relative to NPC tissue (Fig. 2). We first determined whether the diagnostic effect on MAP2-IR varied across the three regions. No region by diagnosis interaction effect on log_e_(MAP2-IR) was observed in our analysis (*F*_2,74_ = 1.7 and *p* = 0.19). Once we had established that any diagnostic effect was common across the regions, we assessed the main effects of both diagnosis and region. Our RM-ANOVA model identified significant effects of both diagnosis (*F*_1,19_ = 31.1 and *p* < 0.001) and region (*F*_2,74_ = 46.4 and *p* < 0.001) on log_e_(MAP2-IR). SZ subjects exhibited lower MAP2-IR, and MAP2-IR tended to increase moving toward rostral cortex (Fig. 3a). Average log_e_(MAP2-IR) deficits between SZ subjects and matched NPC subjects of 17.0% (V1), 22.8% (LIP), and 19.5% (DLPFC) represented linear MAP2-IR deficits of 69.6%, 69.3%, and 56%, respectively. MAP2-IR values are represented in linear scale in Supplementary Fig. 1. The effect of diagnosis was also apparent when MAP2-IR levels were averaged across region within individuals (Fig. 3b). MAP2-IR decrease was observed between most matched pairs of subjects in all regions; MAP2-IR of an NPC subject numerically exceeded MAP2-IR of the paired SZ subject in 15/19 pairs at V1, 17/19 pairs at DLPFC, and 16/20 pairs at LIP (Fig. 3c–e). This was also reflected in matched pair log_e_(MAP2-IR) ratios (SZ:NPC) (Fig. 3f).

**Fig. 4:**
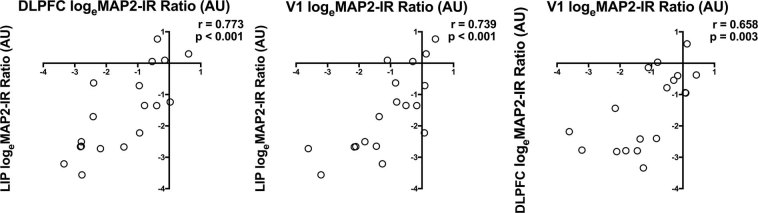
SZ-related MAP2-IR decreases occur across regions on a within-subject basis. Interregional correlation matrix of log_e_(MAP2-IR) pairwise ratios (SZ:NPC). Points in the lower left quadrant indicate that the SZ subject had lower MAP2-IR than the paired NPC subject in both cortical areas. *N* = 18 pairs. AU = arbitrary units

Finally, we examined interregional patterns of log_e_(MAP2-IR) ratios (SZ:NPC). Correlation analysis revealed strong conservation of pairwise ratios across regions, indicating that within a given subject with SZ, MAP2-IR deficits are similar across the three cortical regions examined (Fig. 4).

In addition, the possible confounding effects of several clinical and cohort variables on MAP2-IR were assessed. MAP2-IR was not significantly associated with age, tissue storage time, sex, or postmortem interval (PMI), though it was significantly and positively associated with pH (Table 2). However, incorporating tissue pH as a fixed effect in our statistical model did not diminish the effect of diagnosis (*F*_1,18_ = 23.7, *p* < 0.001). Further, pairwise MAP2-IR differences did not depend significantly on the use of nicotine, antipsychotics, antidepressants, or benzodiazepine/valproic acid (VPA) at time of death (Table 2). Finally, none of the regions exhibited a significant diagnosis by run effect (V1: *F*_4,14_ = 1.09, *p* = 0.40; LIP: *F*_4,14_ = 0.31, *p* = 0.87, DLPFC: *F*_4,14_ = 0.03, *p* > 0.99), indicating that assay run did not contribute to the effect of diagnosis.

Asphyxiation, hypoxia, and ischemia have previously been shown to reduce numbers of MAP2-IR-positive neurons in human neocortex and hippocampus.^19,20^ We examined the effect of these phenomena on MAP2-IR in our cohort using data regarding subjects’ medical conditions, causes of death and identified neuropathologies (Supplementary Table 1). There was a trend for the combined variable Asphyxiation/Hypoxia/Ischemia (AHI) (see “Methods” section) to have been present more frequently in SZ subjects (Table 1). None of the three variables individually associated with MAP2-IR in our cohort; however, the combined variable was associated with decreased MAP2-IR (Table 2). Nevertheless, when the combined variable was entered into our RM-ANOVA model, it was no longer significantly associated with MAP2-IR (*F*_1,19_ = 2.3, *p* = 0.15), whereas the effect of diagnosis remained significant (*F*_1,19_ = 21.3, *p* < 0.001).

**Table 1:** Cohort characteristics

	NPC	Schizophrenia (SZ)
*n*	20	20
Age, mean ± SD	47.7 ± 9.5	45.4 ± 9.3
Sex, M/F	14/6	14/6
pH, mean ± SD	6.7 ± 0.2	6.5 ± 0.3
PMI, hours ± SD	15.5 ± 5.7	14.5 ± 6.1
Storage time, months ± SD	114.1 ± 42.8	113.6 ± 48.9
Nicotine ATOD, Y/N		14/4
Antipsychotic ATOD, Y/N		18/2
Antidepressant ATOD, Y/N		11/9
Benzodiazepine/VPA ATOD, Y/N		5/15
Asphyxiation, Y/N	3/17	8/12
Hypoxia, Y/N	4/16	4/16
Ischemia, Y/N	0/20	5/15
AHI, Y/N	6/14	12/8

Diagnostic groups showed no significant differences in age (*t*_37_ = 0.68, *p* = 0.50), PMI (*t*_37_ = 0.55, *p* = 0.58), storage time (*t*_37_ = 0.14, *p* = 0.89), pH (*t*_34_ = 1.79, *p* = 0.08), nor in frequency of asphyxiation (*X*^2^[1,*N* = 20] = 3.13, *p* = 0.08), ischemia (*X*^2^[1,*N* = 20] = 3.66, *p* = 0.06), hypoxia, or the presence of one or more of these risk variables (AHI; see text) (*X*^2^[1,N = 20] = 3.64, *p* = 0.06). Additional cohort details can be found in Supplementary Table 1

*SD* standard deviation, *PMI* postmortem interval, *M* male, *F* female, *ATOD* at time of death, *AHI* asphyxiation/hypoxia/ischemia (see text)

**Table 2:** Univariate tests of association of MAP2-IR with cohort variables

	Δlog_e_(MAP2-IR)	*F*_1,34_	*p*
Age	−0.03	1.69	0.20
Sex (M = Y, F = N)	0.26	0.22	0.64
PMI	0.01	0.04	0.85
pH	1.96	6.66	0.01
Storage time	0.01	1.81	0.19
		*F*_1,19_	*p*
Asphyxiation	−0.72	1.7	0.21
Hypoxia	−0.74	1.2	0.29
Ischemia	−1.42	4.2	0.06
AHI	−1.23	7.0	0.02
	Δ% Pairwise log_e_(MAP2-IR) change (Y group – N group)	*t*_15_^a^	*p*
Nicotine use ATOD	−4.99	0.55	0.59
		*t*_16_	*p*
Antipsychotic use ATOD	−16.74	1.07	0.30
Antidepressant use ATOD	−2.05	−0.27	0.79
Benzodiazepine/VPA use ATOD	−2.33	−0.28	0.78

Δlog_e_(MAP2-IR) is defined as Y–N group for dichotomous variables, or the slope of each continuous variable on MAP2-IR. AHI is a combined variable indicating the presence of asphyxiation, hypoxia, and/or ischemia (see text and Supplementary Table 1)

*ATOD* at time of death

^a^Nicotine ATOD status was unknown for one subject

## Discussion

We here demonstrate that MAP2-IR deficits are correlated across multiple cortical lobes within individuals with SZ. MAP2-IR loss between SZ subjects and matched NPC subjects was conserved across three cortical regions: DLPFC, LIP, and V1.

Evidence of MAP2-IR reductions in SZ has accumulated so steadily in recent decades that the phenomenon was recently described as a molecular “hallmark” of the disorder.^13^ Quantitative and qualitative decreases in MAP2-IR have been identified in SZ postmortem brain tissue in cortical areas spanning the frontal and temporal lobes.^3–8^ Here we demonstrate significantly lower levels of MAP2-IR across DLPFC, LIP, and V1 within individual subjects with SZ. While patterns of MAP2-IR loss across cortex within individuals with SZ have not been directly investigated prior to this work, several postmortem studies have assayed multiple brain regions in the same subject pool, finding similar IR losses across regions; for instance, Arnold et al.^3^ measured MAP2-IR levels in both entorhinal cortex and subiculum using a single cohort of six SZ-diagnosed subjects (compared with six healthy controls), reporting qualitatively absent MAP2 staining in five and four SZ subjects in each respective area. Similarly, Jones et al.^6^ found significant reductions in layer III MAP2-positive area fraction at Brodmann areas 9 and 32 (36% each) without changes in neuron number, using a single cohort of seven SZ/NPC pairs. These examples of consistent within-cohort reductions in the hippocampal formation and the PFC are consistent with our finding of MAP2-IR reductions that are conserved across cerebral cortex lobes within individuals with SZ.

However, Jones et al.^6^ also reported no significant changes in MAP2 area fraction within V1 of SZ subjects. Moreover, this pattern was recapitulated in a later study^21^ finding a significantly smaller layer III MAP2 area fraction in BA9 and a nonsignificant change in BA17. This contrasts with a significant reduction in MAP2-IR at V1 described in the present work. Differences in both methodology and experimental design might contribute to inconsistent results in this region. The binary thresholding of MAP2-IR employed in area fraction analysis may be insensitive to diagnosis-dependent reductions in IR intensity that are more modest yet retain dendritic morphology. Notably, in our data, V1 exhibited the lowest levels of MAP2-IR among the regions studied for both diagnostic groups (Fig. 3f); this may limit the detectability of MAP2-IR positive area in this region. However, strong correlations with IR reductions in DLPFC and LIP (Fig. 4) as well as our larger cohort size (*n* = 40) lend support to our conclusion that the observed MAP2-IR reduction in V1 is a biologically relevant effect. MAP2-IR changes in SZ parietal cortex, to the best of our knowledge, have never been previously analyzed.

This effect of diagnosis is not driven by effects of clinical or tissue variables, including age, sex, tissue storage time, tissue pH, or PMI. To minimize any possible influence of confounding factors, subject pairs were matched as closely as possible by these parameters (Supplementary Table 1). There were no significant differences in any such variables between diagnostic groups with the exception of tissue pH (Table 1). Reductions in pH have previously been reported for postmortem tissue of SZ subjects and may arise as a consequence of antipsychotic treatment.^22^ Incorporating tissue pH as a fixed effect in our statistical model did not diminish the significant effect of diagnosis, however, indicating that tissue pH alone cannot explain the observed differences in MAP2-IR between diagnostic groups. One prior study, in a rodent model of PMI, found that MAP2-IR was qualitatively reduced after 2 h of PMI, then remained stable through 48 h.^23^ Our finding of no association of PMI with MAP2-IR (Table 2) is consistent with this prior report, as all of our subjects fell into this latter 2–48 h PMI range. The use of various medications at time of death also did not significantly affect MAP2-IR (Table 2), indicating that MAP2-IR loss is not a side effect of medical treatment. We have previously also failed to observe an effect of antipsychotic medications on MAP2-IR in a small nonhuman primate cohort in primary auditory cortex.^7^

Moreover, the MAP2-IR changes reported herein do not appear to reflect decreases in neuron number. Neuron density remains unchanged in SZ frontal, parietal, or occipital neocortex^24^ and has shown no change by diagnosis in BA17 or is increased.^25,26^ It is also unlikely to result from reduced MAP2 mRNA or protein levels. MAP2 mRNA is unaltered in the hippocampus^9^ or layer III pyramidal cells^10^ of SZ subjects, and no change in MAP2 peptide levels was observed by LC-MS in our work in BA41 within subjects in whom MAP2-IR reductions were profound.^7,8^ Various proteomic assays of SZ have failed to observe any alterations in MAP2 protein levels in DLPFC^27–29^ anterior cingulate cortex^30,31^ or hippocampus^32,33^ despite frequent alterations in other cytoskeletal proteins (such as tubulin) in such assays. From this evidence combined, we can reasonably conclude that the observed MAP2-IR reduction is not an artifact of clinical or postmortem confounds, nor a pathology secondary to MAP2 transcript/protein loss, but instead reflects a specific SZ-associated alteration in the MAP2 protein which occurs throughout cerebral cortex.

Though the cause(s) of MAP2-IR reduction in SZ remain unknown, one hypothesis^3,4,7^ posits that lack of antigen binding results from a shift in protein structure precipitated by changes in PTM of MAP2. In particular, MAP2 is known to be heavily regulated by phosphorylation.^34^ We have recently communicated the differential expression of five MAP2 phosphopeptides in SZ, showing that MAP2 phosphorylation state is altered in the disorder.^35^ Interestingly, MAP2 phosphorylation state appears to represent a convergence point for several upstream, SZ-relevant signaling pathways. MAP2 phosphorylation is directly or indirectly modulated by several proteins which are clearly identified by unbiased genomic studies as contributing to SZ risk^36–38^ including MAPK family member MAPK3,^39^ voltage-gated calcium ion channels^40^ and NMDA receptors.^41–43^

Direct alterations to MAP2 structure through aberrant PTMs could go on to affect MAP2 function; the site-specific phosphorylation state of MAP2, for instance, is known to affect its microtubule-binding, -polymerizing, and -stabilizing capabilities^34,44,45^ and therefore could contribute to regulation of the dendritic microtubule cytoskeleton. Indeed, olfactory neuronal precursors derived from individuals with SZ exhibit disorganized microtubule networks as evidenced by βIII-tubulin staining,^46^ implicating aberrant microtubule organization in SZ. In turn, microtubules have become increasingly implicated in dendritic spine development, maintenance, and postsynaptic plasticity.^47–51^ Consistent with these observations, we have noted that SZ-associated reductions in spine density in primary auditory cortex are restricted to individuals with low MAP2-IR.^7,8^ Further understanding the underlying sources of MAP2-IR reductions and their impact on microtubule function, therefore, holds potential as a therapeutic target to rescue SZ-associated abnormalities in synaptic structure.

The generalization of cortical MAP2-IR loss reported herein will be a significant point of consideration in future investigations of MAP2 pathology in SZ. For example, the present findings raise the possibility that the positive correlation between MAP2-IR and dendritic spine density we observed in BA41^7,8^ could exist in other brain areas; indeed, reductions in spine density have previously been reported in SZ DLPFC^52^ and subiculum.^53^ In addition, a widespread pattern of MAP2-IR loss in SZ suggests that this MAP2 modification is a robust biomarker of the disorder which, if causally related to SZ symptoms, may be amenable to therapeutic intervention. Our data, combined with prior studies, suggest that SZ-associated MAP2 alterations might affect numerous cognitive and sensory cortical areas across the brain within a given subject. Further, our failure to observe a significant diagnosis by region interaction effect on MAP2-IR suggests that MAP2-associated pathologies in SZ similarly affect these different cortical areas. As such, interventions which target SZ-associated MAP2 alterations, such as phosphorylation, may be broadly useful in the treatment of SZ. Such intervention, if implemented globally, also may have potential to improve functionality of neural networks spanning multiple cortical areas, such as that involved in visuospatial-working memory, which is impaired in SZ patients.^54,55^

However, if MAP2 can be targeted therapeutically, such treatments are not likely to benefit all SZ-diagnosed patients. In our previous work in primary auditory cortex, we described subsets of subjects with SZ who exhibited “normal” levels of MAP2-IR (higher than the lowest NPC value,^7^ or above the 25th percentile of NPC MAP2-IR^8^), indicating that MAP2 pathology is not present in all individuals with SZ. It is also possible that in some individuals, disruptions in MAP2 activity are to an extent compensated for through the actions of other MAPs, reducing any potential benefit from a MAP2-directed treatment. While MAP2 mRNA/protein deficiency has been shown to result in reduced neurite outgrowth and aberrant microtubule organization both in vitro and in vivo,^56–59^ MAP2 knockout (KO) is nonlethal in the mouse until combined with MAP1b KO.^60^ Conversely, MAP1b KO-associated deficits in axonal length are rescued through MAP2c overexpression,^60^ further indicating overlapping functionality of the two MAPs. Therefore, the careful subcategorization of individuals with SZ based on severity of MAP2 pathology would be necessary to effectively utilize MAP2 as a therapeutic target.

In conclusion, MAP2-IR was assessed in DLPFC, LIP, and V1 of SZ subjects compared with NPC subjects. We found significant diagnosis-dependent reductions in MAP2-IR. The effect was similar across cortical regions within subject pairs. These data provide evidence of a global cortical MAP2-IR decrease in SZ and further elevate the MAP2 protein as a valuable therapeutic target, possibly capable of yielding improvement in multiple symptom domains. However, further studies to characterize the prevalence of MAP2 pathology in SZ, the source of MAP2-IR loss, and the extent, nature, and consequences of functional changes to MAP2 in the disorder (if any) will all be necessary to harness its therapeutic potential.

## Methods

### Human subjects

**Fig. 5:**
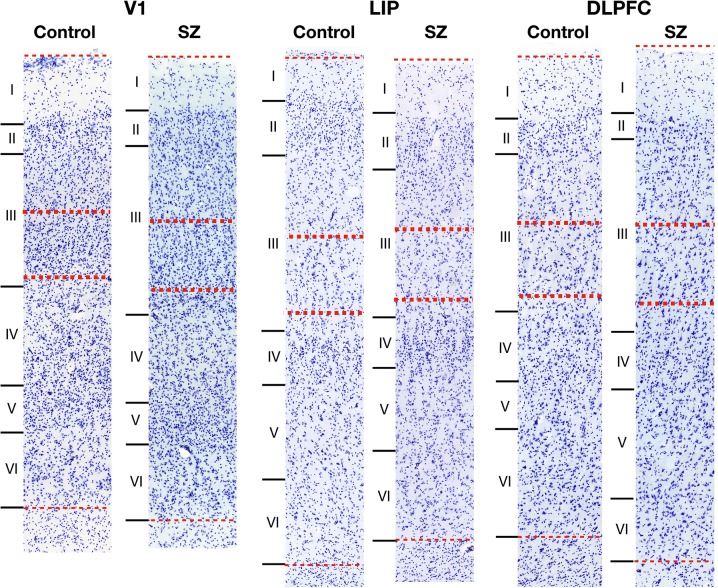
Representative bright-field micrographs of Nissl-stained V1, LIP, and DLPFC. Total cortical thickness from pial surface to white matter (thin dotted lines) was measured and the region to be sampled in deep layer III was approximated as 35–50% of this depth (thick dotted lines). Deep layer III contains many large pyramidal cells in contrast to superficial layer III, which contains small to medium-size pyramidal cells. Cortical layers of V1 are defined by Hässler’s laminar scheme^64^

After receiving consent from next-of-kin, brain tissue was obtained during autopsies at the Allegheny County Office of the Medical Examiner and consensus DSM-IV diagnoses were made by an independent committee of experienced clinicians on the basis of clinical records and interviews with surviving relatives who have expressed written informed consent.^52,61^ These procedures were approved by the Institutional Review Board for Biomedical Research at the University of Pittsburgh and Committee for Oversight of Research and Clinical Training Involving the Dead. Each SZ subject was matched to an NPC subject for sex and as closely as possible for age and PMI. See Table 1 for cohort details. The left hemisphere of each brain was blocked coronally, immersed in 4% paraformaldehyde in phosphate buffer for 48 h, equilibrated in a series of graded sucrose solutions, and stored at −30 °C in an antifreeze solution. Cryostat sections were cut at 40 µm and sections containing V1, DLPFC, and LIP, as identified based on anatomic location (Fig. 1 b–d) and cytoarchitectural features of adjacent (within 400 µm) Nissl-stained sections (Fig. 5), were chosen for staining (one section per region per subject) and stored in antifreeze solution at −30 °C.

### Immunohistochemistry (IHC)

Samples were processed across five IHC runs. All regions of interest from a subject pair were processed in the same run. MAP2-IR was detected as previously described^7,8^ using monoclonal antibody SMI-52 (1:500, Biolegend cat#801801). SMI-52 has been shown to react with mammalian MAP2 both in culture and in fixed sections, robustly labeling the soma and dendritic arbor of neurons in human tissue.^62^ In immunoblot experiments, SMI-52 recognizes all isoforms of MAP2 (MAP2A, MAP2B, MAP2C, and MAP2D).^8,63^

Tissue was rinsed in phosphate buffer (0.1 M in distilled H_2_O) 3 × 8 min. Sections were pre treated at room temperature in 1% NaBH_4_ for 30 min to reduce auto-fluorescence before being rinsed in phosphate-buffered saline (PBS) (8 × 3 min) and incubated in Triton-X (0.3% in PBS) at room temperature for 30 min. Tissue was then incubated for 2.5 h at room temperature in blocking buffer (20% normal goat serum [Jackson Immunoresearch cat#005-000-121], 20% normal human serum [Jackson Immunoresearch cat#009-000-121], 1% BSA, 0.1% Lysine, and 0.1% Glycine in PBS) before overnight incubation (24 h) at 4 °C in incubation solution (5% normal goat serum, 5% normal human serum, 1% BSA, 0.1% Lysine, and 0.1% Glycine in PBS) with SMI-52 antibody. Samples were rinsed in PBS (4 × 30 min) before overnight incubation at 4 °C in incubation solution containing biotinylated goat anti-mouse antibody (1:200, Vector Laboratories cat#BA-9200). In addition, Phalloidin-Alexa Fluor 568 (15 µL/mL, Thermo Scientific cat#A12380) was included in this incubation solution to allow for a MAP2-IR-independent assessment of tissue thickness and *Z*-position within the sections during imaging.

Sections were then rinsed again before overnight incubation with streptavidin-Alexa Fluor 647 (1:500, Thermo Scientific cat#S32357). Finally, samples were rinsed before mounting on gel-coated slides. Tissue was left to dry at least 1 h before rehydration in distilled H_2_O to minimize shrinkage. Samples were coverslipped with Vectashield hard-set H-1400 mounting medium (Vector Laboratories, Burlingame, CA) then left to dry at room temperature overnight before storage at 4 °C until imaging.

### Tissue sampling

All samples were coded to blind the experimenter (RAD) to diagnosis prior to sampling. Cortical deep layer 3 was systematically approximated as 35–50% of total depth from pial surface to white matter based on Nissl-stained histology (Fig. 5). Contours outlining this layer were drawn in Stereo Investigator version 8 (MicroBrightField Inc., Natick, MA). Sites (≥10) of 54.12 µm^2^ within each contour were assigned using a randomly-rotated sampling grid generated in Stereo Investigator, where grid size equaled the square root of the total contour size divided by 10. Tissue thickness was recorded at each sampling site and did not differ between diagnostic groups (*F*_1,1171_ = 0.73, *p* = 0.39).

### Confocal microscopy

Images were taken using a 1.42 numerical aperture ×60 supercorrected oil objective mounted on an Olympus BX51Wl confocal microscope (Olympus, Center Valley, PA) equipped with an Olympus DSU spinning disk, Hamamatsu Orca R2 camera (Hamamatsu, Bridgewater, NJ), MBF CX9000 front-mounted digital camera (MicroBrightField Inc., Natick, MA), BioPrecision2 XYZ motorized stage with linear XYZ encoders (Ludl Electronic Products Ltd, Hawthorne, NY), excitation and emission filter wheels (Ludl Electronic Products Ltd, Hawthorne, NY), Sedat Quad 89000 filter set (Chroma Technology Corp., Bellows Falls, VT), and a Lumen 220 metal halide lamp (Prior Scientific, Rockland, MA). 2D images were taken in Slidebook software version 5.027 at 2 μm below tissue surface as done previously,^7,8^ using 647 nm excitation for MAP2-IR and 405 nm excitation to capture lipofuscin auto-fluorescence. Lipofuscin is a pigment consisting of lysosomal degradation products that accumulates with age and is abundant in postmortem human brain tissue. Exposure times were optimized for the best spread of intensity histogram data in an NPC tissue section and thereafter kept constant across all subjects (100 ms for 647 nm excitation and 500 ms for 405 nm excitation). Subject pairs and their respective regions of interest were always imaged on the same day, in a randomized order. Ten assigned sites were imaged within each contour except when unavailable due to the following exclusions: sites with large auto-fluorescent features such as blood vessels were excluded from analysis, as were tissue sections which sustained significant damage during coverslipping and/or imaging. This resulted in a total of 1175 2D images. For further detail (including total area sampled by subject and region), see Supplementary Table 1.

### Image processing

**Fig. 6:**
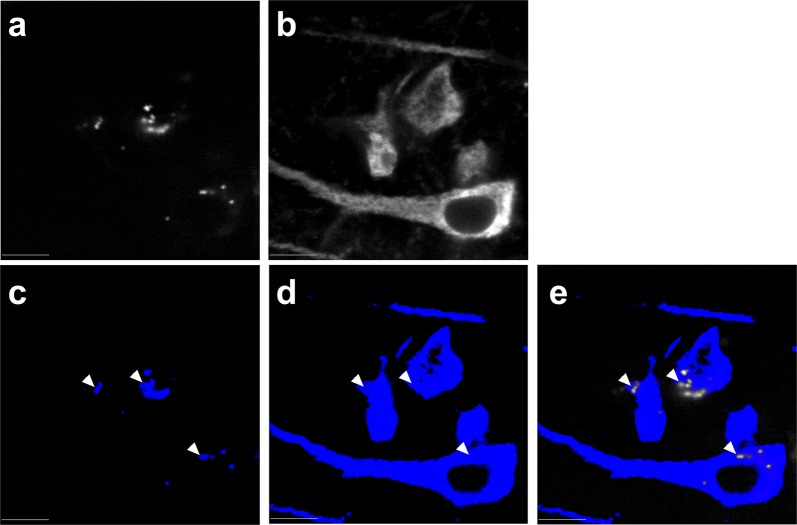
Elimination of lipofuscin auto-fluorescence from the MAP2-IR signal. Lipofuscin (**a**) was imaged by excitation at 405 nm with emissions collected at 668 nm. Signal was subjected to thresholding at a value manually selected from an intensity histogram to derive a binary mask (**c**). Ridler–Calvard thresholding was used to convert 647 channel emissions (**b**) into a mask (**d**), from which the 405 channel mask was subtracted to yield a final mask (**e**) for intensity analysis. Arrowheads indicate sites of mask subtraction. Scale bars = 10 µm

Processing occurred in Slidebook with keystrokes automated by Automation Anywhere software (Automation Anywhere, Denver, CO). 647 channel emissions of the 2D images were masked using threshold segmentation defined by a Ridler–Calvard (RC)-derived value in Slidebook. 405 channel emissions were manually masked and subtracted from the 647 channel RC mask to avoid the confound of lipofuscin auto-fluorescence (Fig. 6). This generated the final mask from which grayscale intensity data was extracted. Averages across sites within each tissue section were calculated to yield a single MAP2-IR value per subject per region for analysis. Camera background was subtracted from the data prior to analysis.

### Statistics

MAP2-IR values were log_e_-transformed for all statistical analyses to achieve a more normal distribution of the data. To analyze diagnostic and region effects on MAP2-IR, a repeated measures analysis of the variance (RM-ANOVA) model of mean log_e_(MAP2-IR) value was used, with pair, diagnosis, region, and diagnosis by region as fixed effects and subject as a random effect to account for the multiple measurements per subject. To assess the possible confounding effects of cohort variables including age, sex, PMI, tissue pH, and tissue storage time, another RM-ANOVA model for mean log_e_(MAP2-IR) values was used, containing region, main effects of each cohort variable, and region by cohort variable interaction as fixed effects and subject as a random effect. For IHC run, an ANOVA model with pair and diagnosis by run as fixed effects was used to analyze each region separately. To more robustly examine the possible confounding effect of tissue pH, a third RM-ANOVA model was used for the mean log_e_(MAP2-IR) value in each region, including pair, region, diagnosis, and tissue pH as fixed effects and subject as a random effect. Further, effects of hypoxia and ischemia—both of which have been previously shown to impact MAP2 immunolabeling^19,20^—were assessed by assigning all subjects a binary score to indicate the presence or absence of (1) a cause of death involving asphyxiation, (2) a medical condition likely to induce hypoxia, (3) a neuropathology indicative of ischemic injury, or (4) a combined variable, asphyxiation/hypoxia/ischemia (AHI), indicating the presence of any of the three (Supplementary Table 1). The effects of these variables on MAP2-IR were assessed using RM-ANOVA models, where each model contained region and pair as fixed effects and subject as a random effect. In addition, Chi square tests of independence were performed to test for changes in frequency of these risk factors between diagnostic groups. To test for interregional conservation of MAP2-IR loss within pairs, a Pearson correlation coefficient matrix was calculated between the pairwise log_e_(MAP2-IR) difference (=log[MAP2-IR_SZ_/MAP2-IR_NPC_]) of each region. For dichotomous variables, including nicotine, antipsychotic, antidepressant, and benzodiazepine/VPA use at time of death, equal variance *t*-tests were used to test equality across the dichotomy of the pairwise percent log_e_(MAP2-IR) difference (=100 × [log(MAP2-IR_SZ_/MAP2-IR_NPC_)]) between groups.

### Reporting summary

Further information on research design is available in the Nature Research Reporting Summary linked to this article.

## Supplementary information

Supplementary Material

Reporting Summary

## Data Availability

The data that support the findings of this study are available from the corresponding author upon reasonable request.

## Supplementary information

**Supplementary information** accompanies the paper on the *npj Schizophrenia* website (10.1038/s41537-019-0081-0).
