# Supplementary material for: MAP2 immunoreactivity deficit is conserved across the cerebral cortex within individuals with schizophrenia
Source: NPJ Schizophr. 2019 Aug 28;5:13. doi: 10.1038/s41537-019-0081-0 (PMC6713711; doi:10.1038/s41537-019-0081-0)
Supplement: Supplementary file 1 — Supplementary Material [file 41537_2019_81_MOESM1_ESM.pdf]

Pair	Subject	Diagnosis	Sex	Race	Age (years)	PMI (hours)	Storage time (months)	pH	Nicotine ATOD	Antipsychotic ATOD	Antidepressant ATOD
1	1583	C	M	W	58	19.1	39.37	6.7			
	1686	S	M	B	56	14.1	22.6	6.2	Yes	Yes	Yes
2	1372	C	M	W	37	20.5	79.8	6.6			
	1581	S	M	W	32	18.4	39.57	6.8	Yes	Yes	Yes
3	1307	C	M	B	32	4.8	89.53	6.7			
	10024	S	M	B	37	6	98.93	6.1	No	No	No
4	10003	C	M	W	49	21.2	107.97	6.5			
	1088	S	M	W	49	21.5	119.47	6.5	Yes	Yes	Yes
5	567	C	F	W	46	15	207.23	6.7			
	537	S	F	W	37	14.5	212.3	6.7	Unknown	No	No
6	1247	C	F	W	58	22.7	100.13	6.4			
	1240	S	F	B	50	22.9	100.83	6.3	Yes	Yes	No
7	1092	C	F	B	40	16.6	119.07	6.8			
	1178	S	F	B	37	18.9	110.47	6.1	Yes	Yes	No
8	681	C	M	W	51	11.6	190.9	7.2			
	640	S	M	W	49	5.2	196	6.9	Unknown	Yes	Yes
9	1191	C	M	B	59	19.4	108.37	6.2			
	1263	S	M	W	62	22.7	97.77	7.1	Yes	Yes	Yes
10	1391	C	F	W	51	7.8	75.47	6.6			
	1189	S	F	W	47	14.4	108.63	6.4	Yes	Yes	Yes
11	1326	C	M	W	58	16.4	86.17	6.7			
	1314	S	M	W	50	11	88.77	6.2	No	Yes	Yes
12	1284	C	M	W	55	6.4	94.53	6.8			
	1188	S	M	W	58	7.7	108.87	6.2	Yes	Yes	No
13	1099	C	F	W	24	9.1	118.07	6.5			
	10023	S	F	B	25	20.1	99.4	6.7	No	Yes	No
14	1159	C	M	W	51	16.7	111.97	6.5			
	1296	S	M	W	48	7.8	92.23	6.5	Yes	Yes	Yes
15	1386	C	M	W	46	21.2	76.17	6.7			
	1420	S	M	W	47	23.4	69.63	6.7	Yes	Yes	Yes
16	546	C	F	W	37	23.5	211.2	6.7			
	587	S	F	B	38	17.8	204.03	7	Yes	Yes	No
17	1268	C	M	B	49	19.9	96.87	7.1			
	1230	S	M	W	50	16.9	102.47	6.6	Yes	Yes	Yes
18	852	C	M	W	54	8	159.2	6.8			
	781	S	M	B	52	8	174.2	6.7	Yes	Yes	Yes
19	1122	C	M	W	55	15.4	115.47	6.7			
	1105	S	M	W	53	7.9	117.53	6.2	Yes	Yes	No
20	1047	C	M	W	43	13.8	125.63	6.6			
	1209	S	M	W	35	9.1	106.3	6.5	No	Yes	No

Supplementary Table 1. Cohort characteristics by subject. C = control; S = schizophrenia; W = white; B = black; ATOD = at time of death.

Benzodiazepine/VPA ATOD	Cause of Death	Manner of Death
	Blunt Force Trauma (Trunk) caused by MVA with tree	Accidental
Yes	ASCVD	Natural
	Asphyxiation (Compression of Upper Torso)	Accidental
No	ASCVD	Natural
	Hypertensive Cardiomyopathy	Natural
No	Critical Coronary Atherosclerosis (Multiple Subacute Pulmonary Thromboemboli)	Natural
	Blunt Force Trauma (Multiple)	Accidental
No	Combined Drug Toxicity	Accidental
	Mitral Valve Prolapse	Natural
No	Asphyxiation by Hanging	Suicide
	ASCVD (With Renal Failure)	Natural
No	Hypertensive Heart Disease	Natural
	Mitral Valve Prolapse	Natural
Yes	Pulmonary Thromboembolism (DVT)	Natural
	Hypertrophic Cardiomyopathy	Natural
No	Pulmonary Embolism (DVT)	Natural
	Atherosclerotic and Hypertensive Heart Disease	Natural
No	Asphyxiation Due to Airway Obstruction (food bolus)	Accidental
	ASCVD	Natural
Yes	Combined Drug Overdose	Suicide
	ASCVD (IDDM contributory)	Natural
No	ASCVD	Natural
	ASCVD	Natural
No	ASCVD	Natural
	Cardiomyopathy, NOS	Natural
Yes	Drowning	Suicide
	Hypertensive Heart Disease	Natural
No	Necrotizing Pneumonia (Abscess formations in R Lung)	Natural
	Hypertensive Cardiovascular Disease and Atherosclerotic Coronary Artery Disease	Natural
No	Trauma -Jump (Lacerated L heart atrium; skull fracture)	Suicide
	ASCVD	Natural
Yes	Hypertensive Heart Disease	Natural
	Atherosclerotic and Hypertensive Heart Disease	Natural
No	Anoxic Encephalopathy (Doxepin Toxicity)	Suicide
	Cardiac Tamponade (rupture of ascending aortic aneurysm)	Natural
No	Peritonitis (Displacement of G Tube)	Accidental
	Cardiac Tamponade (ruptured dissecting aortic aneurysm)	Natural
No	ASCVD	Natural
	ASCVD	Natural
No	Diphenhydramine Overdose	Suicide

Supplementary Table 1 (continued)

Medical Diagnoses
Emphasema; Abnormal EEG
COPD; Hypertension; ASCVD
Hypertension; Sleep Apnea; Obesity; Dilated Cardiomyopathy
Hypertension
CHI (age 44) with LOC, "blood on brain" (work accident)
Hypertension
Coronary Artery Disease
LOC in MVA age 34;
Hypertension; CVA & TIA (nothing found on neuropath); Congestive Heart Failure; End-Stage Renal Disease (on dialysis)
Hypertension; Asthma; Morbid Obesity
Congenital Heart Defect
Obesity
Left Ventricular Hypertrophy
Asthma, Sleep Apnea, Congestive Heart Failure, Hypertension
COPD; Hypertension; ASCVD
Hypertension
CHI with LOC(age 44): frontal subdural hydromas, 2 week anterograde amnesia, & respiratory failure; possible TIAs; Pneumonia; Hypertension; Asthma, Chronic Bronchitis & Tracheal Bronchitis with MRSA
Hypertension; Obesity
Hypertension
Hypertension; Obesity
Multiple CHI (ages 3 & 8, no LOC); Asthma; ASCVD
Hypertension
Hypertension
Hypertension
Hypertension
Anoxic seizures; CHI with brain abscess (age 20 - motorcycle accident); traumatic subarachnoid hemorrhage with communicating hydrocephalus; post-traumatic CSF fistula (age 22)
COPD, Hypertension, EEG - diffuse slowing
Hypertension; Bronchitis
Hypertension; Seizures
Hypertension
Bronchitis

Supplementary Table 1 (continued)

Neuropathology	Asphyxiation	Hypoxia	Ischemia	Asphyxiation/Hypoxia/Ischemia
	Yes	No	No	Yes
Ventriculomegaly, mild, bilateral frontal horns of lateral ventricles	No	Yes	No	Yes
	Yes	Yes	No	Yes
Enlarged ventricles for age, mild to moderate	Yes	No	No	Yes
	No	Yes	No	Yes
	Yes	No	No	Yes
	No	No	No	No
Beta A4 Amyloid deposits, moderate, w/o other pathological changes associated with AD	No	No	Yes	Yes
	No	No	No	No
	Yes	No	No	Yes
	No	Yes	No	Yes
	No	No	No	No
	No	No	No	No
	No	No	No	No
	No	No	No	No
Increased Sulci width L temporal lobe	No	No	No	No
	No	Yes	No	Yes
Contusion (encephalomalacia, focal) in the R temporal tip (old); AD changes consistent with age	Yes	Yes	Yes	Yes
	Yes	No	No	Yes
	No	No	No	No
	No	No	No	No
	No	No	No	No
	No	No	No	No
AD pathology evident - meets CERAD criteria for definite AD if there is a clinical history	No	No	Yes	Yes
	No	No	No	No
	Yes	No	No	Yes
	No	No	No	No
Hippocampal Atrophy, Mild	Yes	No	No	Yes
	No	No	No	No
Subarachnoid Hemorrhage, focal, base of brain	Yes	No	No	Yes
	No	No	No	No
	No	No	No	No
	No	No	No	No
Ischemic injury in hippocampus, acute, mild; Cerebellar infarct, subacute, microscopic	Yes	Yes	Yes	Yes
Atherosclerotic plaques; multiple large, Circle of Willis	No	No	No	No
Mild atrophy; cerebral infarct, frontal, remote	No	Yes	Yes	Yes
	No	No	No	No
	No	No	No	No
Choroid Plexis cyst, R lateral ventricle	No	No	No	No
	No	No	No	No

Supplementary Table 1 (continued)

Sites Imaged (total area imaged [um2])		
V1	LIP	DLPFC
10 (521.2)	10 (521.2)	10 (521.2)
10 (521.2)	10 (521.2)	10 (521.2)
10 (521.2)	10 (521.2)	10 (521.2)
10 (521.2)	10 (521.2)	10 (521.2)
10 (521.2)	10 (521.2)	10 (521.2)
10 (521.2)	10 (521.2)	10 (521.2)
10 (521.2)	10 (521.2)	10 (521.2)
10 (521.2)	10 (521.2)	10 (521.2)
**0 (0)**	10 (521.2)	10 (521.2)
10 (521.2)	10 (521.2)	10 (521.2)
10 (521.2)	10 (521.2)	10 (521.2)
10 (521.2)	10 (521.2)	10 (521.2)
10 (521.2)	10 (521.2)	10 (521.2)
10 (521.2)	10 (521.2)	10 (521.2)
10 (521.2)	10 (521.2)	10 (521.2)
10 (521.2)	10 (521.2)	10 (521.2)
10 (521.2)	10 (521.2)	10 (521.2)
10 (521.2)	10 (521.2)	10 (521.2)
10 (521.2)	10 (521.2)	10 (521.2)
10 (521.2)	10 (521.2)	10 (521.2)
10 (521.2)	10 (521.2)	10 (521.2)
10 (521.2)	10 (521.2)	10 (521.2)
10 (521.2)	10 (521.2)	10 (521.2)
**8 (416.96)**	10 (521.2)	**0 (0)**
10 (521.2)	10 (521.2)	10 (521.2)
10 (521.2)	10 (521.2)	10 (521.2)
10 (521.2)	10 (521.2)	10 (521.2)
10 (521.2)	10 (521.2)	10 (521.2)
10 (521.2)	10 (521.2)	10 (521.2)
10 (521.2)	10 (521.2)	10 (521.2)
**9 (469.08)**	10 (521.2)	10 (521.2)
10 (521.2)	10 (521.2)	10 (521.2)
**9 (469.08)**	10 (521.2)	10 (521.2)
10 (521.2)	10 (521.2)	10 (521.2)
10 (521.2)	10 (521.2)	10 (521.2)
10 (521.2)	10 (521.2)	**9 (469.08)**
10 (521.2)	10 (521.2)	10 (521.2)
10 (521.2)	10 (521.2)	10 (521.2)
10 (521.2)	10 (521.2)	10 (521.2)
10 (521.2)	10 (521.2)	10 (521.2)

Supplementary Table 1 (continued)

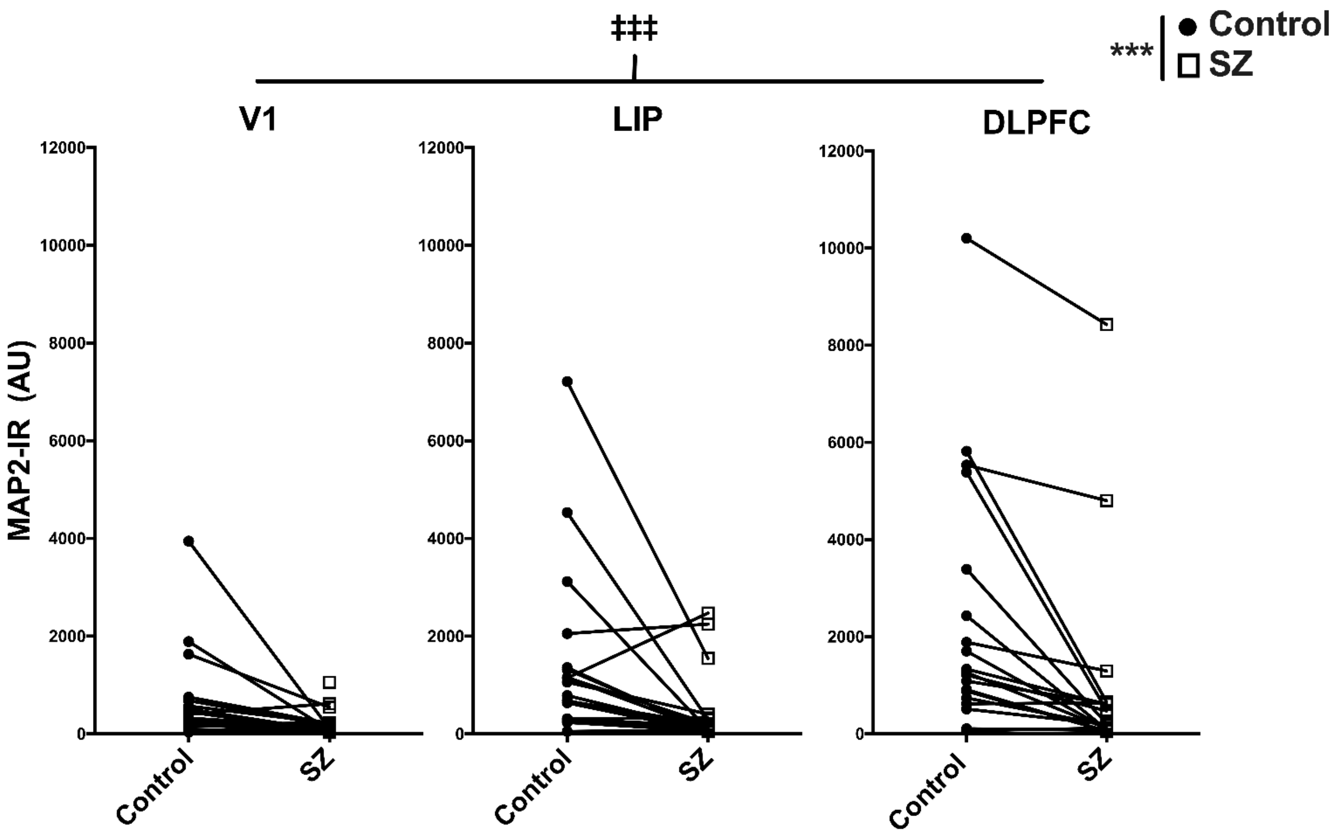

Supplementary Fig. 1 MAP2-IR is reduced uniformly in V1, LIP and DLPFC of SZ patients relative to matched NPC subjects. Before-after plots of MAP2-IR levels in paired subjects. N = V1: 19 pairs; LIP: 20 pairs; DLPFC: 19 pairs. *** p < 0.001 (diagnosis effect). ‡‡‡ p < 0.001 (region effect). AU = arbitrary units.
